# Simvastatin Inhibits Renal Cancer Cell Growth and Metastasis via AKT/mTOR, ERK and JAK2/STAT3 Pathway

**DOI:** 10.1371/journal.pone.0062823

**Published:** 2013-05-17

**Authors:** Zhiqing Fang, Yueqing Tang, Juanjuan Fang, Zunlin Zhou, Zhaoquan Xing, Zhaoxin Guo, Xiaoyu Guo, Weichang Wang, Wei Jiao, Zhonghua Xu, Zhaoxu Liu

**Affiliations:** 1 Department of Urology, Qilu Hospital of Shandong University, Ji'nan, Shandong, China; 2 The Key Laboratory of Cardiovascular Remodeling and Function Research, Chinese Ministry of Education and Chinese Ministry of Public Health, Qilu Hospital of Shandong University, Ji'nan, Shandong, China; 3 Department of Anesthesiology, Wucheng People's Hospital, Wucheng, Shandong, China; 4 School of Nursing, Shandong University, Jinan, Shandong, China; The University of Hong Kong, Hong Kong

## Abstract

Renal cell carcinoma (RCC) is the most lethal type of genitourinary cancer due to its occult onset and resistance to chemotherapy and radiation. Recently, accumulating evidence has suggested stains, inhibitors of 3-hydroxy-3-methyl glutaryl coenzyme A (HMG-CoA) reductase, were associated with the risk reduction of cancer. In the present study, we aimed to investigate the potential effects of simvastatin on RCC cells and the underlying mechanisms by which simvastatin exerted its actions. With cell viability, colony formation, and flow cytometric apoptosis assays, we found that simvastatin potently suppressed cell growth of A498 and 786-O cells in a time- and dose- dependent manner. Consistently, the xenograft model performed in nude mice exhibited reduced tumor growth with simvastatin treatment. In addition, the inhibitory effects of simvastatin on migration and invasion were also observed in *vitro*. Mechanically, we presented that simvastatin could suppress the proliferation and motility of RCC cells via inhibiting the phosphorylation of AKT, mTOR, and ERK in a time- and dose- dependent manner. Further investigation of the underlying mechanism revealed simvastatin could exert the anti-tumor effects by suppressing IL-6-induced phosphorylation of JAK2 and STAT3. In conclusion, these findings suggested that simvastatin-induced apoptosis and its anti-metastasis activity in RCC cells were accompanied by inhibition of AKT/mTOR, ERK, and JAK2/STAT3 pathways, which imply that simvastatin may be a potential therapeutic agent for the treatment of RCC patients.

## Introduction

Renal cell carcinoma (RCC) is the most common type of renal cancer, accounting for approximately 90–95% kidney neoplasms [1]. Worldwide, mortality due to RCC has exceeded 100,000 patients each year [2]. About 25–30% of patients develop metastases at diagnosis of RCC [3], with survival ≥5 years ranging from 5% to 10%, and overall median survival of less than one year [4], [5]. Surgical intervention is the primary treatment for localized RCC, but alone it has limited benefit in patients with aggressive disease [1]. In addition, traditional cytotoxic chemotherapy and immunotherapy have failed to demonstrate a benefit in patients in the adjuvant setting [6]. Recent years have seen a rapid development of molecular targeted therapy [7]. Amongst the first-line targeted therapies, sunitinib and temsirolimus are the most representative, which can block the signaling pathway of multiple receptor tyrosine kinases (RTKs) and mammalian target of rapamycin (mTOR) respectively [8], [9]. Nevertheless, none of the interventions would be considered cost-effective at a willingness-to-pay threshold of 30,000 pounds per quality-adjusted life-year [10]. Thus, there is a great demand for treatments that can prolong survival without greatly increasing costs or eroding the quality of patients' lives.

Statins (or 3-hydroxy-3-methylglutaryl coenzyme A [HMG-CoA] reductase inhibitors) are a group of drugs, which are structural analogues of HMG-CoA that inhibit conversion of HMG-CoA to mevalonate [11], [12]. Beyond their cholesterol-reducing properties, statins exhibit numerous pleiotropic effects, including anti-inflammation and immunomodulation [13], [14], reduction in the risk for various forms of dementia [15], and a decrease in proteinuria and the progression of kidney disease [16], [17]. Of significance, emerging evidence revealed that statins could exhibit antineoplastic effects in a variety of cancer cells [18], including prostate cancer [19]–[22], breast cancer [23]–[25], hepatic cancer [26], [27] and colon cancer [28], [29]. Encouragingly, a retrospective nested case-control study that involved 500,000 veterans reported a protective role of statins against the development of RCC [30]. However, the precise effect of simvastatin against RCC cells and the underlying molecular mechanisms have not been well established.

In the present work, our data establish the growth inhibitory and pro-apoptotic effects of simvastatin on RCC cells both in *vitro* and in *vivo*. Meanwhile, we also find that simvastatin can potently decrease the migration and invasion of RCC cells. Further investigation of the underlying mechanisms indicates AKT/mTOR, ERK and JAK2/SAT3 pathways play key roles in these actions. Our findings suggest the clinical implications of simvastatin in the treatment of RCC patients.

## Materials and Methods

### Ethics statement

The animal experimental protocol complied with the Animal Management Rules of the Chinese Ministry of Health (Document No. 55, 2001) and was approved by Animal Care and Use Committee of Shandong University. Pathogen-free BALB/c nude mice (weighing 19±2 g, SPF grade, certificate SCXK2011-0012) of 4–5 week old were purchased from Department of Laboratory Animal Science of Peking University (Beijing, China). All animals were maintained at the key Laboratory of Cardiovascular Remodeling and Function Research in Qilu Hospital of Shandong University.

### Cell lines and reagents

Human A498 and 786-O cell lines were obtained from American Type Culture Collection (ATCC) and cultured in DMEM high Glucose medium (HyClone) with 10% fetal bovine serum (FBS) under the conditions of 5% CO2 at 37°C. Simvastatin (sodium salt, C25H39O6·Na) was purchased from Sigma (St. Louis, MO, USA), which was activated according to the manufacturer' instructions. Human IL-6 was purchased from R&D Systems (Minneapolis, MN, USA). Antibodies against phospho-mTOR, mTOR, phospho-AKT(Ser473), AKT, phospho-JAK2, JAK2, phospho- STAT3 (Tyr705 and Ser727), STAT3, PARP, GAPDH, and HRP-conjugated goat anti-rabbit and anti-mouse IgG were obtained from Cell Signaling Technology (Beverly, MA, USA). Antibodies against casepas-3, bax, bcl-2 and survivin were obtained from Immuno-way (Newark, DE, USA). Antibodies against phospho-ERK, ERK were obtained from Anbo (San Francisco, CA, USA).

### Cell viability assay

Cell viability was determined by 3-(4, 5-dimethylthiazol-2-yl)-2, 5-diphenyltetrazolium bromide (MTT) assay. Briefly, A498 (2×10^3^ cells/well) and 786-O (1×10^3^ cells/well) in 100 µl medium were seeded in 96 well plates. After 12 hours, the medium in each well was replaced with the medium containing different concentrations of simvastatin and the plate was incubated for 48, 72 and 96 hours. Subsequently, 20 µl of MTT (5 mg/ml) was added into each well. After incubation at 37°C for 4 h, the supernatant was removed and 200 µl of DMSO was added to each well. After the precipitation was fully dissolved, the absorbance values were determined with the Microplate Reader (Bio-Rad, Hercules, CA, USA).

### Effect of simvastatin on cell morphology

When A498 and 786-O cells reached 70% of confluence, the cells were washed with phosphate-buffered saline (PBS) and then exposed to 16 µM simvastatin for 48 hours. The morphology of treated cells was observed under microscope and photomicrographs were taken with Olympus digital camera.

### Clone formation assay

A498 and 786-O cells were seeded at density of 1×10^3^ cells/well in 6-well plates. After incubation overnight, each well was added of simvastatin (8 and 16 µM) and incubated for 24 hours. Then the medium was replaced with complete medium without simvastatin and incubated under the conditions of 5% CO_2_ and 37°C for two weeks. When visible clones formed on the plates, the incubation was terminated. The clones were washed with PBS and then fixed with methanol and stained with 0.1% crystal violet for 30 min. The colony containing more than 50 cells was counted under a microscope.

### *In vitro* scratch assay

*In vitro* scratch assay was performed as described previously [31]. A498 and 786-O cells were seeded in 24-well plates. After incubation for 24 hours, each well was manually scratched with a 200 µl pipette tip, washed with PBS three times and incubated at 37°C with simvastatin (8 and 16 µM). The scratch area was photographed 18 hours later. The distance between two cell edges were analyzed by ImageJ software.

### Invasion and migration assay

The transwell system (24 wells, 8 µm pore size with poly-carbonate membrane; Corning Costar, Lowell, MA, USA) coated with 2 mg/ml Matrigel (BD Biosciences) was used for the in *vitro* invasion assays. A total of 5×10^5^ cells were suspended in 100 µl serum-free medium and were added to the upper chambers. DMEM containing 20% FBS and simvastatin (8 and 16 µM) was then added to the lower chamber. After 24 hours, cells remaining on the upper chambers were removed with a cotton swab whereas the cells attaching to the lower surface were fixed with methanol and stained with 0.1% crystal violet. The number of cells migrated to the lower side was counted in five randomly fields under a light microscope. The cell number was counted and analyzed statistically.

For migration assay, the cells were seeded in upper chambers without coated Matrigel. The rest of assay was performed as the invasion assay. After 18 hours, the cells on lower surface were also counted in five randomly fields, then the cell number was analyzed statistically.

### Apoptosis assay

This assay was performed to detect cell apoptosis with an Annexin V-FITC Apoptosis Detection Kit (BD Biosciences, San Jose, CA). In brief, harvested cells were resuspended in 100 µl of the binding buffer to achieve a concentration of 1×10^6^/mL. Then, 5 µl Annexin V-FITC and 5 µl propidium iodide (PI, 20 µg/mL) were added and the tubes were incubated for 15 min at room temperature in dark. Finally, binding buffer (400 µl) was added to each reaction tube and the cells were analyzed by flow cytometry. The data was analyzed by WinMDI V2.9 software (The Scripps Research Institute, San Diego, CA, USA).

### RNA interference and transient transfection

Small interfering RNA (siRNA) targeting human AKT, ERK1/2 and STAT3 were obtained from Cell Signaling Technology (Beverly, MA, USA). A498 cells (2×10^5^ cells/well in 6-well plates) were transfected with AKT, ERK1/2 and STAT3 using Lipofectamine 2000 (Invitrogen) according to the manufacturer's instructions respectively. After transfection, the cells were incubated for 24 h and then treated with simvastatin (8 µM) for MTT, migration, invasion and western blotting assays.

### Western blot analysis

Cells were collected and lysed in RIPA buffer in the presence of protease inhibitors. Protein (50 µg) was separated by SDS-PAGE and transferred onto a PVDF membrane using a wet transfer apparatus (Bio-Rad, Hercules, CA, USA). Membranes were blocked with 5% non-fat milk and incubated overnight at 4°C with the primary antibodies, followed by incubation with the secondary antibodies labeled with horseradish peroxidase. Protein bands were visualized with enhanced chemiluminescence (Millipore). Protein levels were detected using chemiluminescence reader ImageQuant LAS4000 (GE, USA). Protein levels were analyzed by ImageJ software.

### Tumor xenograft model

In brief, a total of 5×10^6^ of A498 cells were mixed with Matrigel and then injected subcutaneously in the flank of nude mice. The mice were randomly divided into two groups (10 of each group). Then mice were given of simvastatin at dose of 5 mg/kg/d by oral gavage for 5 weeks. Control mice were given the same volume of normal saline. Tumor volume and mice weight was measured every week. All of the mice were killed 50 days after inoculation of the cancer cells and the tumors were collected.

### Terminal deoxynucleotidyl transferase dUTP nick end labeling (TUNEL) assay

Xenograft tumors were formalin-fixed, paraffin-embedded and then sliced into 6-µm section for TUNEL assay to identify the apoptotic cells. TUNEL Apoptosis Assay kit (Beoytime, Beijing, China) was used to stain apoptotic cells. These cells were visualized with red fluorescent under a fluorescence microscope (Olympus).

### Statistical analysis

The student's two-tailed t-test was used to determine statistical differences between treatment and control values. Differences were considered statistical significant when p<0.05. All data are presented as the mean ± SD of three independent experiments.

## Results

### Simvastatin inhibits cell proliferation of renal cancer cells

**Figure 1:**
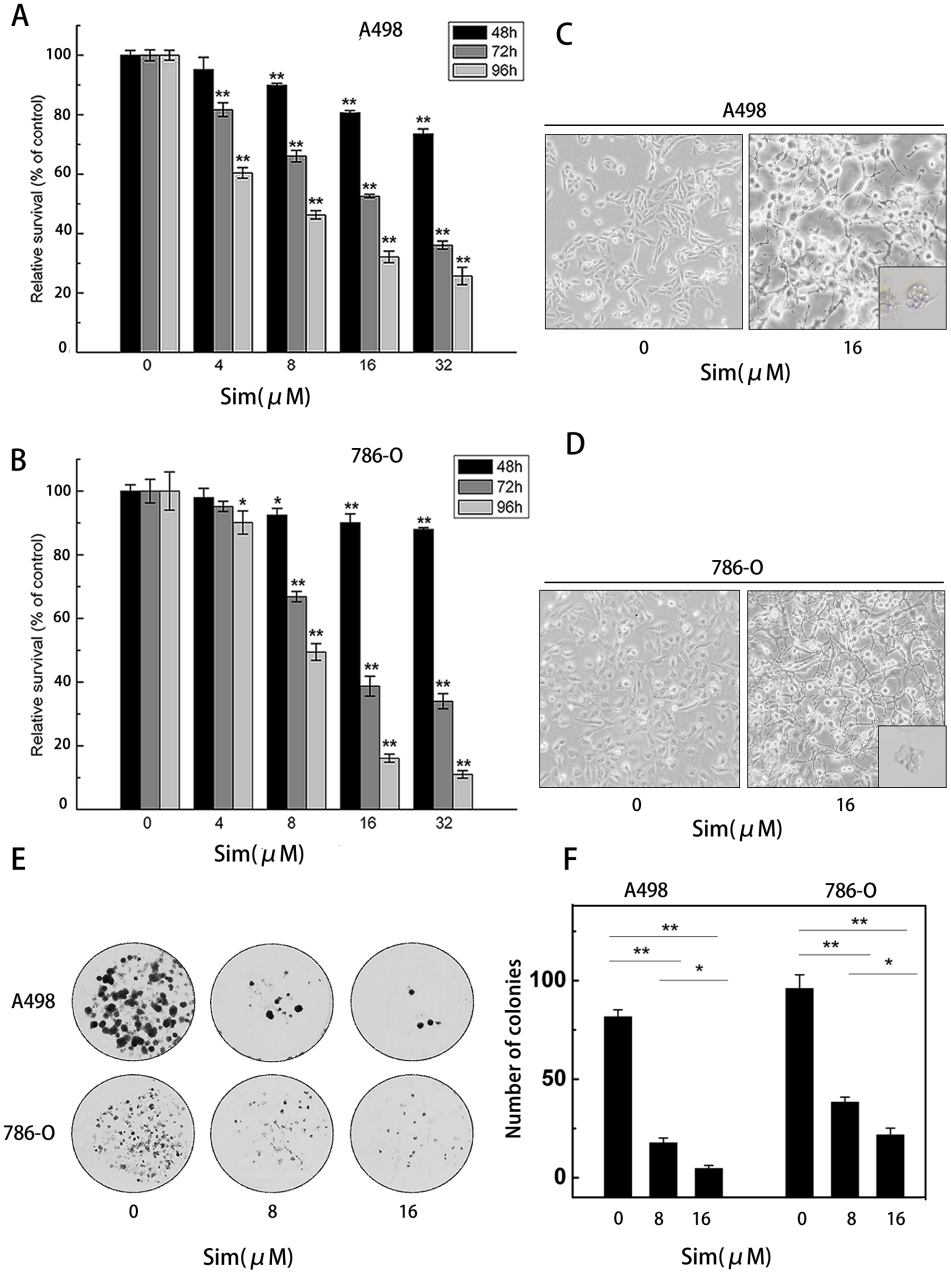
Simvastatin suppressed cell viability of A498 and 786-O cells. The effect of simvastatin on cell viability was measured by MTT assay. (A) A498 cells and (B) 786-O cells were treated with simvastatin for 48, 72 and 96 h. Simvastatin significantly inhibited cell viability of both cell lines in a dose-and time-dependent manner. Morphological changes of A498 and 786-O cells induced by simvastatin were displayed. Images of (C) A498 and (D) 786-O cells before and after addition of simvastatin (16 µm) were taken at 48 h. After treated with simvastatin for 48 h, apoptotic bodies in (C) A498 and (D) 786-O cells can be observed by optical microscope. (E) Inhibitory effect of simvastatin on colony formation. (F) The colony number was counted under microscope and colony is defined to consist of at least 50 cells. The results represent as mean ± SD of three independent experiments and the corresponding standard error. *P<0.05; **P<0.01.

To study the effects of simvastatin on the proliferation of RCC cells, A498 and 786-O cells were exposed to different concentrations of simvastatin for 48, 72 and 96 h in MTT assay. Consequently, simvastatin significantly inhibited the proliferation of A498 and 786-O cells in a time- and dose-dependent manner (*P<0.05, **P<0.01) (Fig. 1A and 1B). The viability of A498 and 786-O cells was reduced to 52.6% and 38.8% after treatment with simvastatin (16 µM) for 72 hours, with the IC50 of 18.434±1.26 µM and 16.311±1.21 µM, respectively.

### Effect of simvastatin on cell morphology of renal cancer cells

Consistent with the previous findings [32], we also observed a two-phased response in tumor cells to simvastatin treatment. The early phase saw a dramatic change in cell morphology within the first 6 h to 24 h. The later phase of the response occurred between 24 h to 72 h, which involved the loss of plasma membrane integrity. Morphology change of A498 and 786-O cells after treatment with simvastatin (16 µM) for 48 hours was shown in Fig. 1C and 1D. As represented, simvastatin caused cells to retract their processes and lose plasma membrane integrity. Shrinkage of the central cell body around the nuclei was observed at the plasma membrane, indicating apoptosis was happening in these cells. The apoptotic body was clearly visible after treatment with simvastatin (16 µM) for 48 h.

### Simvastatin inhibits colony formation in renal cancer cells

We also examined the effects of simvastatin on cell colony formation of the RCC cells. Our study showed that simvastatin inhibited the colony formation of A498 and 786-O cells in a dose-dependent manner (Fig. 1E). The colony number was significantly decreased after RCC cells treated with simvastatin (8 and 16 µM), compared with the control cells (*P<0.05, **P<0.01) (Fig. 1F).

### Simvastatin induces apoptosis in renal cancer cells

**Figure 2:**
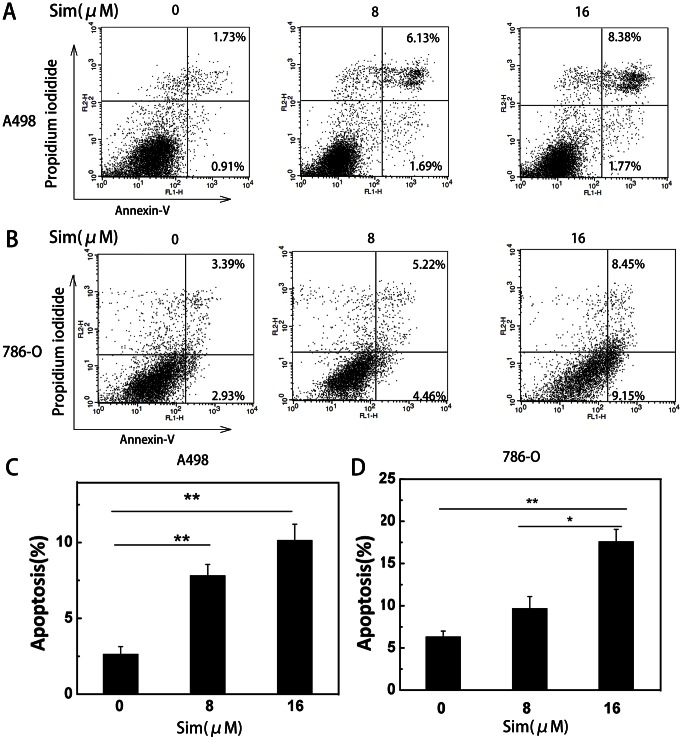
Simvastatin induced dose-dependent apoptosis in A498 and 786-O cells. (A) A498 and (B) 786-O cells were treated with simvastatin (0, 8 and 16 µM) for 48 hours and stained with FITC-annexinV and PI. The percentage of surviving cells was shown in the lower left quadrant; the percentage of early stage of apoptosis and late stage of apoptosis cells were shown in the lower right and upper right quadrants, respectively. (C, D) The quantification of apoptosis induced by simvastatin was calculated. Data is presented as mean ± SD of three independent experiments. * P<0.05, ** P<0.01.

To verify and quantify the apoptotic cells induced by simvastatin, we used Annexin V-conjugated Alexa Fluor 488 and propidium iodide staining to analyze the percentage of apoptotic cells. The percentages of early and later apoptotic cells were shown in the lower right (LR) and upper right (UR) quadrant of the histograms respectively (Fig. 2A and 2B). The total percentage of apoptotic cells (UR+LR) increased from 2.64% in non-simvastatin treated A498 cells to 7.82% and 10.15% in simvastatin-treated cells (8 and 16 µM, respectively) after 48 hours (*P<0.05, **P<0.01) (Fig. 2C). We found the similar phenomenon in 786-O cells, and the total percentage of apoptotic cells was increased from 6.32% to 9.68% and 17.6% (*P<0.05, **P<0.01) (Fig. 2D). Treatment of A498 and 786-O cells with 8 and 16 µM simvastatin for 48 hours induced apoptosis in both cell lines, and it was in a dose-dependent manner. The significant induction of apoptosis after simvastatin treatment indicated its anti-cancer effect on RCC cells.

### Migration and invasion are inhibited by simvastatin in renal cancer cells

**Figure 3:**
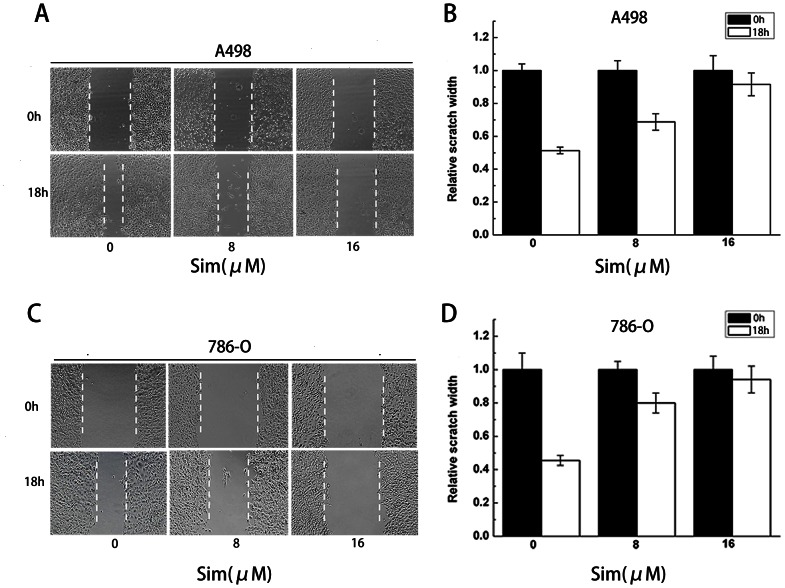
The scratch assay showed the effect of simvastatin on cell migration. (A) Scratch assay of A498 cells treated with 0, 8 and 16 µm of simvastatin. (B) The migration inhibition was transformed to the percentage of the initial distance between the two edges. The simvastatin-treated A498 cells showed a lower rate of wound closure than the control cells. (C) Scratch assay of 786-O cells treated with 0, 8 and 16 µm of simvastatin. (D) The simvastatin-treated 786-O cells showed a lower rate of wound closure than the control cells.

**Figure 4:**
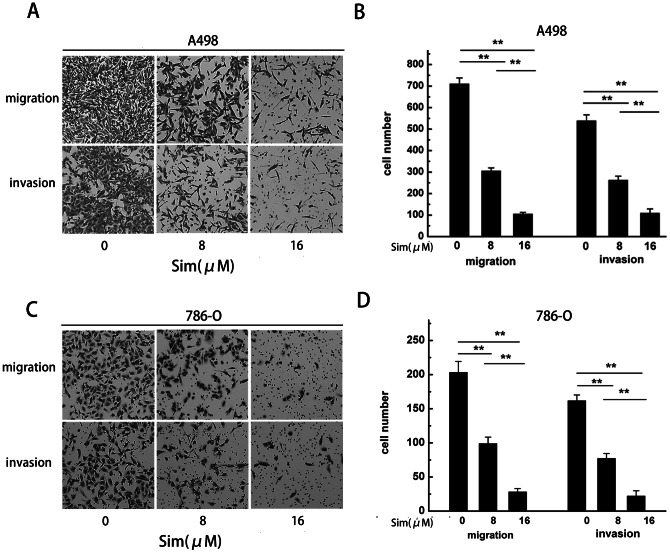
Cell migration and invasion ability were inhibited by increasing concentrations of simvastatin. (A) Treatment with 0, 8 and 16 µm of simvastatin showed inhibited migration and invasion of A498 cells. (B) The number of A498 cells that successfully migrated and invaded was counted. (C) Migration and invasion of 786-O cells was inhibited after treatment with different concentrations of simvastatin. (D) The decreased number of 786-O cells indicated the great inhibitory effect of simvastatin on cell mobility. Data is presented as mean ± SD of three independent experiments. * *P*<0.05, ** *P*<0.01.

The scratch assay was implemented to detect the effect of simvastatin on the migration of renal cancer cell. As shown in Fig. 3A and 3B, migration of A498 cells was restrained by simvastatin in a dose-dependent manner. And the similar effect was also observed in 786-O cells (Fig. 3C and 3D). To further test the influence of simvastatin on cell migration and invasion, A498 cells treated with the increasing concentration of simvastatin were applied to the transwell migration and Matrigel-underpinning transwell invasion assays. Our result showed that simvastatin could significantly inhibit renal cancer cell migration and invasion (*P<0.05, **P<0.01) (Fig. 4A and 4B) in a dose-dependent manner. We found the same effect of simvastatin in 786-O cells (*P<0.05, **P<0.01) (Fig. 4C and 4D).

### Simvastatin inhibits tumor growth and induce tumor cells apoptosis in a xenograft model

**Figure 5:**
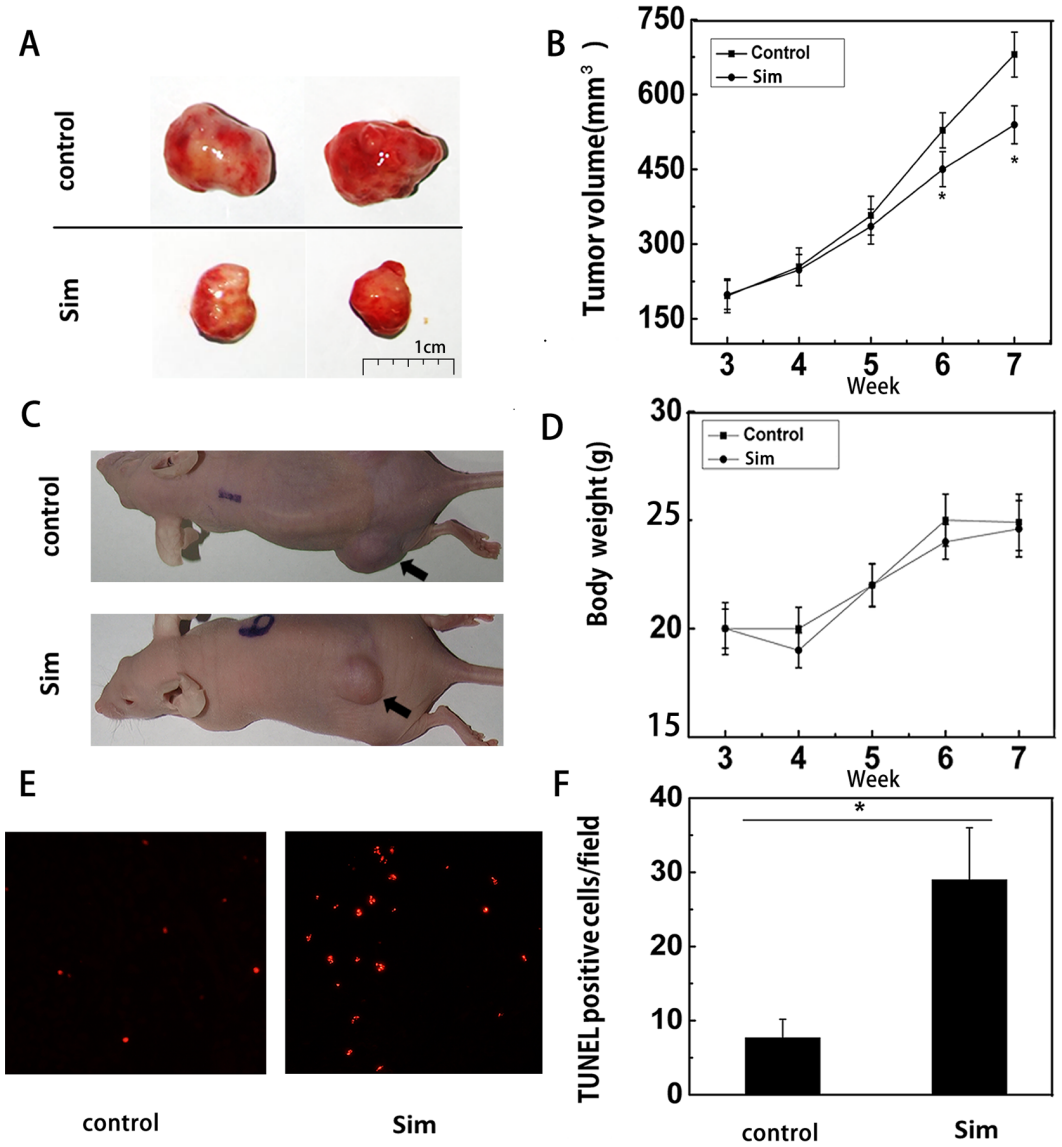
Simvastatin inhibited the growth and induced cell apoptosis in RCC tumor xenografts. Images of the excised tumors (A) and the nude mice (C) were taken from the control and treatment group. The arrows point to the xenografts. (B) Graphs representing the average tumor volumes of A498 xenografts treated with or without simvastatin. (D) Body weight curve of nude mice bearing A498 tumors treated with simvastatin. (E) Representative TUNEL staining (red fluoresence) of A498 renal cancer xenografts. (F) Bar graph showing quantification of the TUNEL positive A498 cells in tumor xenogafts. Data are presented as mean ± SD, *p<0.05.

To determine whether simvastatin inhibits tumor growth in *vivo*, A498 cells (5×10^6^) were injected subcutaneously into each flank of nude mice. Tumor growth inhibition was distinct in mice treated with simvastatin at 5 mg/kg/d, compared with mice treated with PBS (*P<0.05) (Fig. 5A, 5B and 5C). Furthermore, there was no significant toxicity to mice treated with simvastatin (5 mg/kg/d) by assessing mice weight of 2 groups (Fig. 5D). To gain insight whether simvastatin could induce apoptosis of RCC cells in *vivo*, paraffin sections of A498 tumor xenografts from nude mice were applied to the terminal deoxynucleotidyl transferase dUTP nick end labeling (TUNEL) assay. The increased number of TUNEL-positive cells clearly demonstrated that the simvastatin could induce apoptosis of RCC in *vivo* (*p<0.05) (Fig. 5E and 5F).

### Effects of simvastatin on expression of cell apoptosis-related proteins

**Figure 6:**
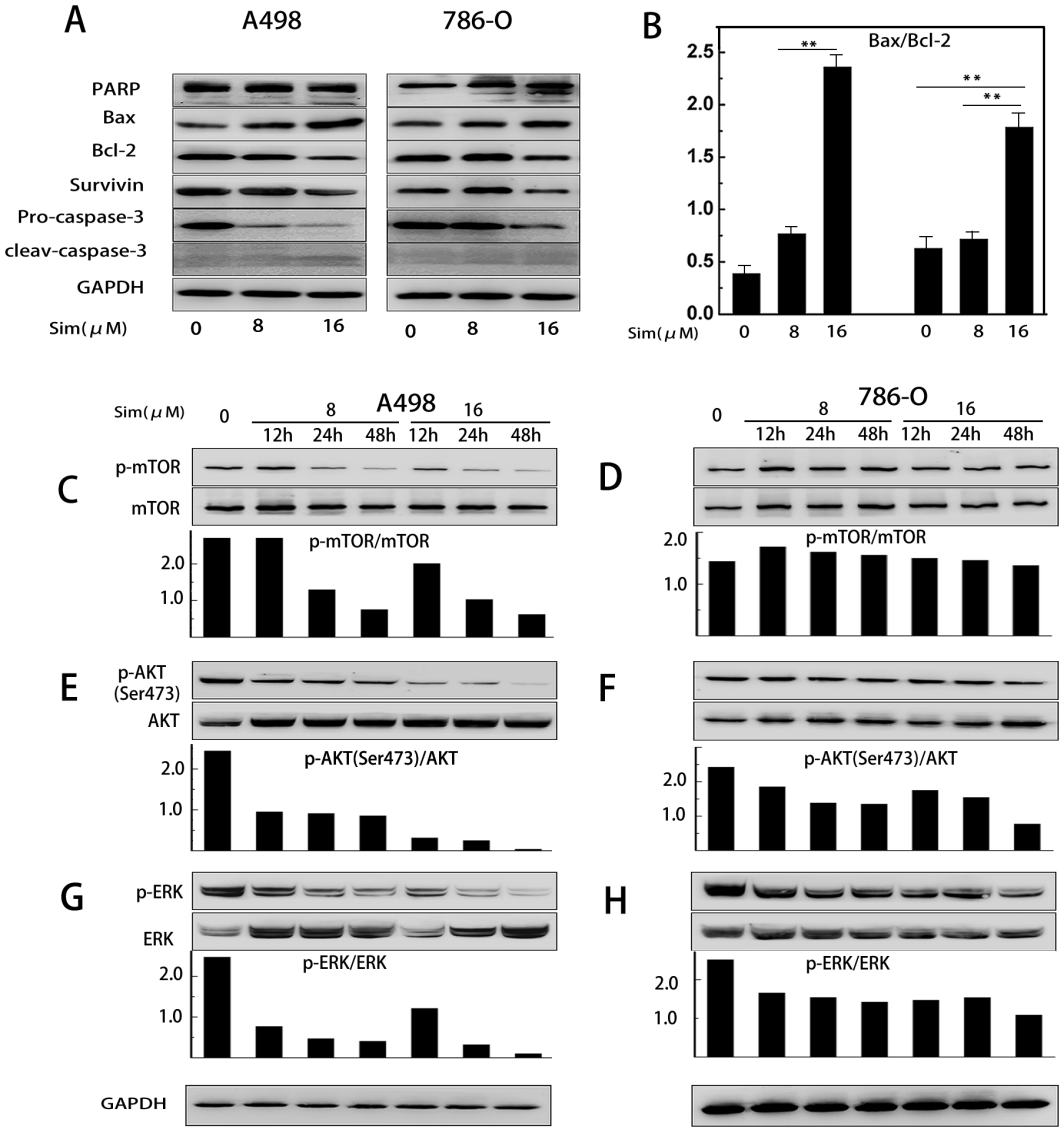
Effects of simvastatin on the protein levels of Bcl-2, Bax, caspase 3, PARP, mTOR, AKT, and ERK in A498 and 786-O cells. (A) A498 and 786-O cells were assayed for Bcl-2, Bax, full length caspase 3 and cleaved caspase 3, full length PARP and cleaved PARP by western blotting analysis with GAPDH as a control. (B) Bax/Bcl-2 ratios of A498 and 786-O cells. The densitometry value of each band was determined with ImageJ. Data was presented as mean ± SD of three independent experiments. * P<0.05, ** P<0.01. (C–H) The levels of mTOR, AKT, ERK and their phosphorylated forms were analyzed by western blotting. Quantitation of the p-mTOR/mTOR, p-AKT/AKT and p-ERK/ERK ratio was determined by densitometry analysis.

The expression of pro-apoptotic protein Bax has been often associated with the increased apoptosis, while the anti-apoptotic protein Bcl-2 has been associated with the inhibition of apoptosis in target cells [33], [34]. After treated with increased concentrations of simvastatin for 48 h, we detected the expression of Bax and Bcl-2 in RCC cells. Consistent with the increased apoptosis in A498 and 786-O, the level of Bax was elevated while the level of Bcl-2 was decreased (Fig. 6A). The ratio of Bax/Bcl-2 protein level is the decisive factor to transmit the apoptosis signal. By comparing the intensity of their bands, we found the ratio of Bax/Bcl-2 was increased in a dose-dependent manner (*P<0.05, **P<0.01) (Fig. 6B). With the elevated ratio of Bax/Bcl-2, the downstream casepase-3 for apoptosis was activated. As is shown, the expression of survivin and pro-caspase-3 was decreased, while the expression of cleaved caspase-3 and cleavage of PARP was elevated.

### Simvastatin inhibits proliferation and metastasis of RCC cells via AKT/mTOR and ERK pathway

Western blotting was used to detect the underlying mechanism by which simvastatin exerted its actions. mTOR is frequently dysregulated in cancer cells, which is under research as a potential target for RCC patients [35]. AKT, as the upstream of mTOR, plays a critical role in the proliferation, survival and motility of cancer cells and it can directly phosphorylate mTOR [36]. Therefore, we investigate the effect of simvastatin on regulation of AKT/mTOR pathway. After A498 and 786-O cells were treated with simvastatin (8 and 16 µM) for 24 and 48 hours, the phosphorylation levels of AKT and mTOR were effectively suppressed. The ratios of p-AKT/AKT as well as p-mTOR/mTOR were also decreased in a time- and dose- dependent manner (Fig. 6C, 6D, 6E and 6F). ERK, which is frequency aberrantly activated in cancer cells, promotes cancer cell proliferation and metastasis [37]. Then we tested the effects of simvastatin on ERK activation, and found simvastatin inhibited phosphorylation of ERK in a time- and dose- dependent manner in both A498 and 786-O cells (Fig. 6G and 6H).

**Figure 7:**
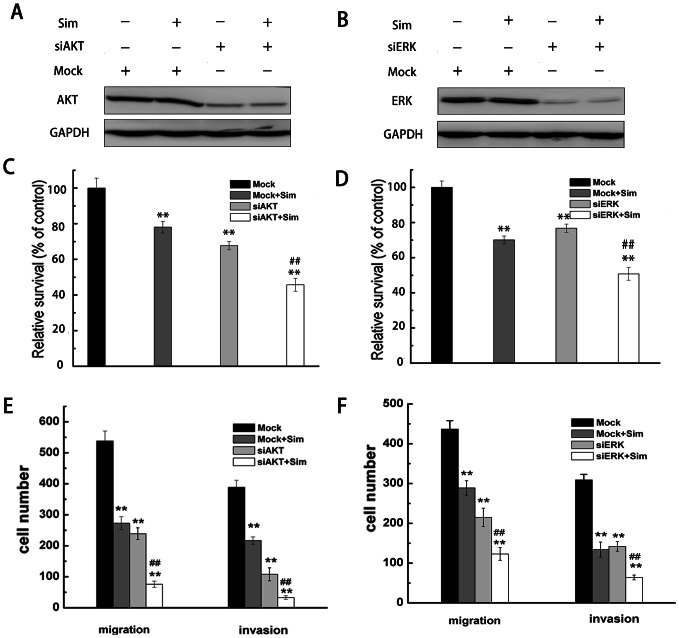
Simvastatin depressed proliferation and metastasis of A498 cells that were transfected with AKT or ERK siRNA compared with the control cells (Mock). (A, B) A498 cells were transfected and treated with simvastatin (8 µM), and the levels of AKT and ERK were analyzed by western blotting with GAPDH as a control. After transfected with AKT or ERK siRNA, A498 cells were incubated in the absence or presence of simvastatin (8 µM) for 48 h. The cell viability was measured by MTT assay (C, D), and cell migration and invasion was measured by transwell assay (E, F). * p<0.05 or ** p<0.01, compared with the untreated cells (Mock). # p<0.05 or ## p<0.01, compared with the cells transfected with AKT or ERK siRNA.

To investigate the role of AKT and ERK in proliferation and mobility of RCC cells, we used siRNA to knockdown AKT and ERK1/2 gene expression in A498 cells. As shown in Fig. 7A and 7B, the western blotting analysis indicated a significant reduction in protein levels of AKT and ERK1/2 in the cells transfected with AKT and ERK1/2 campared to the cells transfected with mock siRNA. Next, we evaluated simvastatin-induced apoptosis and its anti-metastasis activity in A498 cells which were tranfected with AKT or ERK1/2 siRNA. The results showed that knockdown of AKT or ERK1/2 significantly suppressed cell proliferation, migration and invasion (Fig. 7C, 7D, 7E and 7F), indicating that AKT and ERK1/2 play important roles in proliferation, migration and invasion in RCC cells. In addition, simvastatin significantly inhibited the proliferation and motility in AKT or ERK1/2 knockdown cells, which suggest that inhibition of the AKT or ERK signaling pathway could enhance the anti-cancer effect of simvastatin.

### Simvastatin inhibits proliferation and metastasis of RCC cells via IL-6 induced JAK2/STAT3 pathway

**Figure 8:**
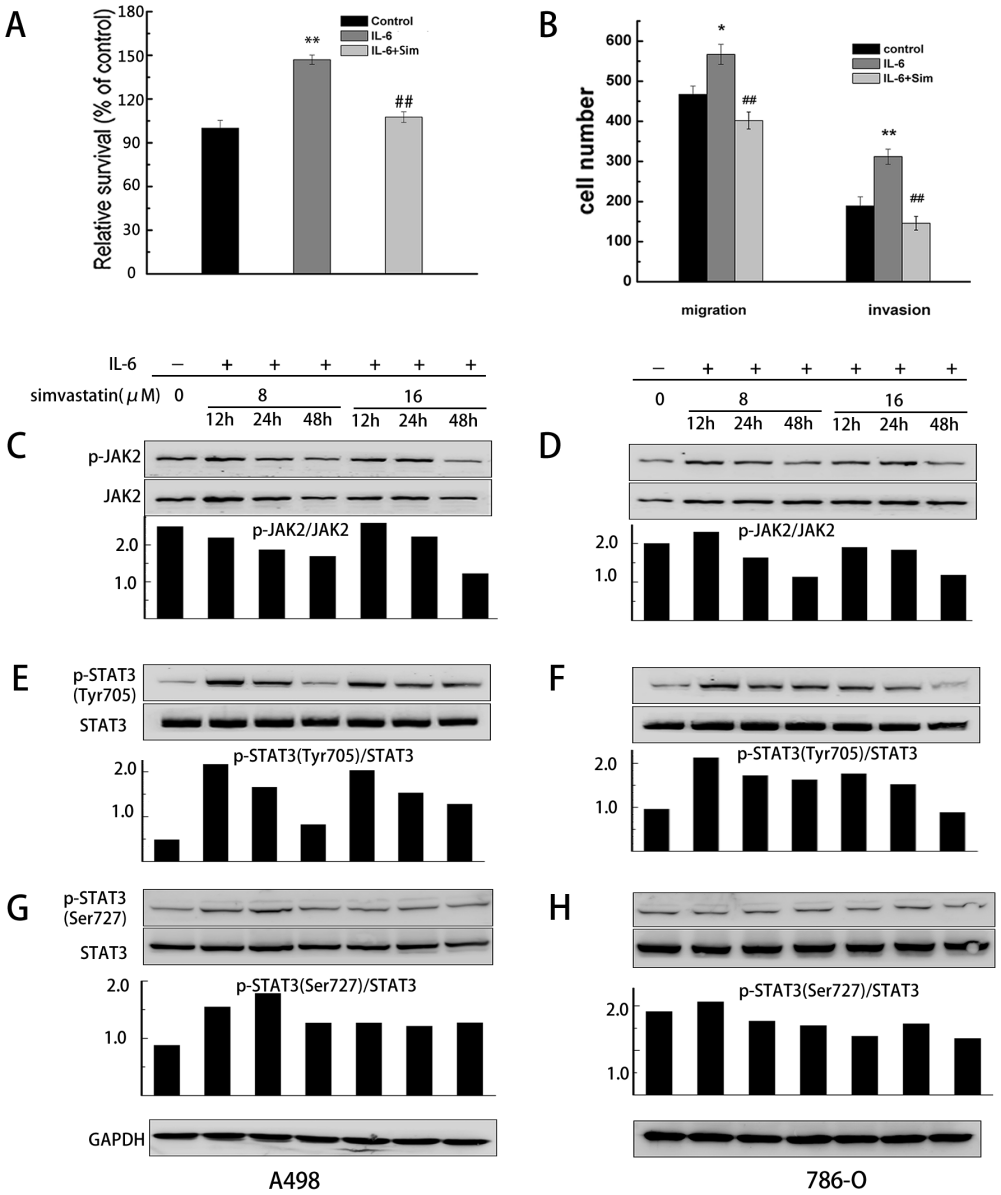
Simvastatin inhibited IL-6 induced proliferation, migration and invasion of A498 cells via inhibition of JAK2/STAT3 pathway. (A, B) A498 cells were treated with IL-6 (10 ng/ml) for 48 h, and cell vitality and motility was estimated using the MTT and transwell assay respectively. Each bar represents the mean±SD of three independent experiments. *p<0.05 or ** p<0.01, compared with cells treated without IL-6. (C–H) Simvastatin inhibited the phosphorylation of Jak2, STAT3 (Tyr705) and STAT3 (Ser727) in renal cancer cells in a dose- and time-dependent manner. Quantitative analysis of the p-JAK2/JAK2, p-STAT3 (Tyr705)/STAT3 and p-STAT3 (Ser727)/STAT3 ratio was displayed in each lower panel.

Previously, statins have been reported to exert pleiotropic effects on cellular signalings and functions involved in inflammation [38]. Thus, we speculate whether simvastatin exerts tumor suppressive effect via modulating the tumor-promoting inflammation. Accumulating evidence has reported the pro-inflammatory JAK2/STAT3 pathway plays an critical role in cancer metastasis, apoptosis and angiogenesis [39]. Notably, IL-6 is a cytokine that can activate JAK2/STAT3 signaling in cancer cells, which has an important effect on oncogenesis [40]. To evaluate whether IL-6 could induce proliferation and metastasis of RCC cancer cells, we serum-starved A498 cells for 12 h and then cultured them in the absence or presence of IL-6 for 48 h. Then cell viability was measured by MTT assay, while cell metastasis ability was measured by transwell assay. We found that IL-6 could significantly induce proliferation and metastasis of A498 cells. In addition, IL-6 induced proliferation and metastasis in A498 cells could be suppressed by simvastatin (8 µM) (Fig. 8A and 8B).

Next, we investigate whether simvastatin inhibits the phosphorylation of JAK2 and STAT3 induced by IL-6 in RCC cells. A498 and 786-O cells were pre-treated with simvastatin (8 and 16 µM) for 12, 24 and 48 hours, and then these cells were incubated with IL-6 (10 ng/ml) for 10 min. Western blotting analyses showed that simvastatin significantly inhibited IL-6 induced phosphorylation of JAK2 and STAT3 at both Tyr705 and Ser727 site (Fig. 8C–8H).

**Figure 9:**
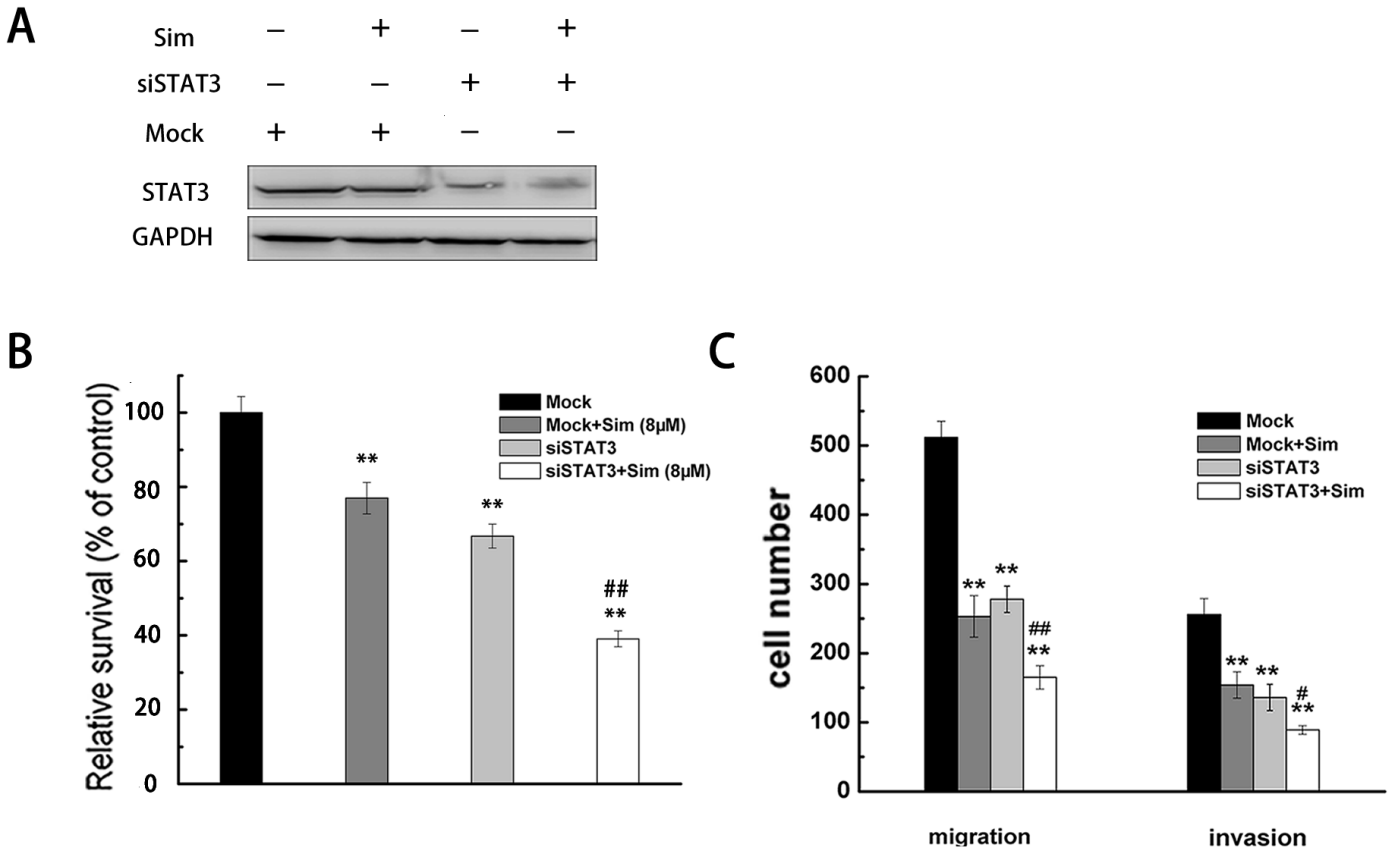
Simvastatin suppressed proliferation and metastasis of A498 cells that were transfected with STAT3 siRNA. (A) A498 cells were transfected with STAT3 siRNA and treated with simvastatin (8 µM), and the levels of STAT3 was analyzed by western blotting with GAPDH as a control. (B, C) After transfected with STAT3 siRNA, A498 cells were incubated in the absence or presence of simvastatin (8 µM) for 48 h. The cell viability was measured by MTT assay, (B), and cell migration and invasion was measured by transwell assay (C). * p<0.05 or ** p<0.01, compared with the untreated cells (Mock). # p<0.05 or ## p<0.01, compared with the cells transfected with STAT3 siRNA.

To assess whether STAT3 is involved in simvastatin-induced apoptosis and anti-metastasis of RCC cells. We applied siRNA to knockdown STAT3 gene expression in A498 cells. After transfected with STAT3 siRNA or control oligonucleotides, the A498 cells were treated with simvastatin (8 µM) or control. As shown in Fig. 9A, knockdown of STAT3 significantly suppressed the proliferation, migration and invasion of A498 cells (Fig. 9B and 9C), indicating that STAT3 plays an important role in proliferation and motility in A498 cells. In addition, combined treatment with simvastatin (8 µM) and STAT3 siRNA decreased cell viability and motility of A498 cells, compared to the cells treated with STAT3 siRNA alone. Therefore, these results suggested that IL-6 induced JAK2/STAT3 pathway was associated with survival and metastasis in RCC cells, and inhibition of IL-6 induced JAK2/STAT3 signaling pathway could sensitize RCC cells to simvastatin treatment.

### Simvastatin inhibits phosphorylation of AKT, ERK and STAT3 in xenograft tumors

**Figure 10:**
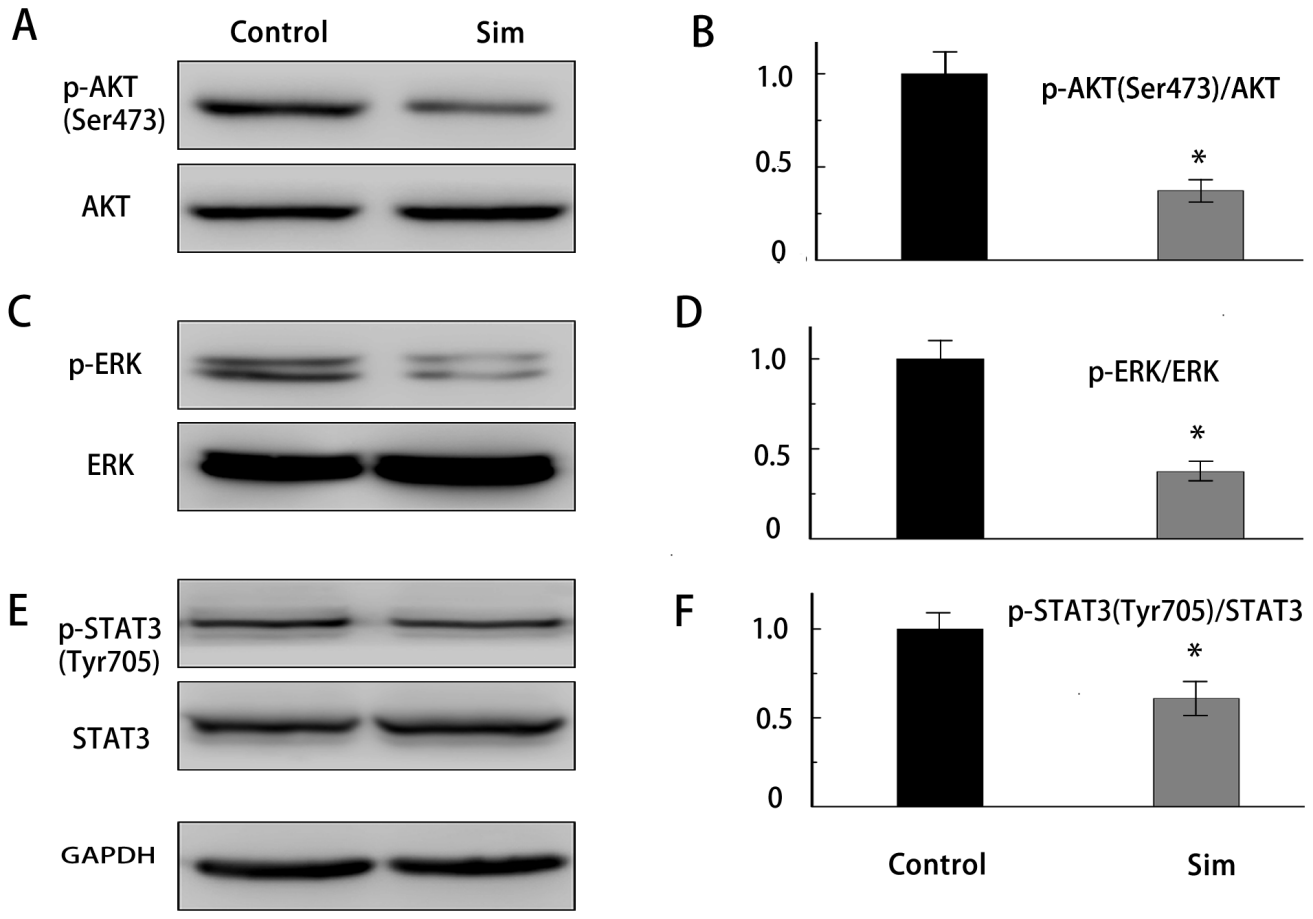
Anti-tumor effects of simvastatin on A498 tumor xenograft were associated with the inhibition of AKT, ERK and STAT3 activity. Western blotting analysis for the phosphorylated AKT, ERK and STAT3 levels in tumor xenograft collected from nude mice treated with or without simvastatin. Quantitation of p-AKT/AKT, p-ERK/ERK and p-STAT3/STAT3 ratio was determined by densitometry analysis. *P<0.05 compared with control.

To determine whether the effect of simvastatin on the growth of A498 tumor xenograft involves the inhibition of AKT, ERK1/2 and STAT3 activity. Western blotting analysis of AKT, ERK1/2 and STAT3 activation indicated that the phosphorylation levels of AKT, ERK1/2 and STAT3 were effectively suppressed in A498 xenograft after treatment with simvastatin in *vivo* (Fig. 10). Overall, our study indicated that simvastatin could inhibit renal tumor growth in *vivo* involving the inhibition of AKT, ERK1/2 and STAT3 activity.

## Discussion

Stains are inhibitors of HMG-CoA reductase, which are widely used in the treatment of lipid disorders, especially hypercholesterolemia [41]. Recently, numerous experimental data have shown that statins exhibit anti-tumor effects against various cancer cells of different origins [42]. Clinical trials evaluating statins either as a single agent or in combination with other agents have also been conducted, and safety of statins has been documented extensively [42]–[45]. The statin family consists of various members, including lovastatin, simvastatin, fluvastatin, pravastatin, atorvastatin and so on [46]. Of these drugs, pravastatin failed to induced any effect in cancer cells [32], whereas other stains could inhibit the proliferation of a wide variety of cancer cells [42]. The observed differences in anti-tumor potential of these stains may be due to their different physicochemical properties. Specifically, their hydrophilic or lipophilic nature is of significance since variations in lipophilicity closely correlated with statins' potential to cross the cellular membrane [42], [47]. Considering the lipophilic nature of simvastatin [21], we aim to investigate its potential effects on renal cancer cells.

In the present study, we showed that simvastatin could potently inhibit renal cancer cell proliferation and mobility in *vitro*. Accordingly, we observed the growth inhibitory and pro-apoptotic effect of simvastatin on renal cancer cells with xenograft model. Furthermore, we examined the apoptotic markers after exposure of different concentrations of simvastatin. The results indicated that simvastatin could induce cleavage of caspase-3 and PARP, down-regulate the pro-apoptotic survivin and Bax as well as up-regulate the anti-apoptotic Bcl-2. Cell cycle arrest was reported to contribute to the growth suppression of statins in cancer cells; however, we find no significant effect of simvastatin on cell cycle distribution in RCC cells (data not shown).

Molecular mechanisms by which statins elicit anti-tumor effects have been a recent focus of investigation. Recent studies have implicated ERK1/2 signaling pathways as mediators of statin-dependent pro-apoptotic effects [48]. It has been reported that fluvastatin could suppress the proliferation of human pancreatic cancer MIAPaCa-2 cells and inhibit the phosphorylation of ERK [49]. It has also been reported that lovastatin could inhibit cell growth by inhibiting the ERK activation [50]. We therefore examined the change of ERK phosphorylation caused by the administration of simvastatin. Consistent with these results, we showed that the levels of phosphorylated ERK were remarkably decreased both *in vitro* and *in vivo*. To determine whether simvastatin played its anti-tumor effects via ERK pathway, we applied siRNA to knockdown ERK expression. After transfected with siRNA of ERK, the cell viability and motility were significantly decreased. These results demonstrated that knockdown of ERK could sensitize renal cancer cells to simvastatin-induced anti-cancer effect.

To clarify the signaling pathways underlying simvastatin-mediated responses in renal cancer cells, we further examined the effect of simvastatin on the activation of the AKT/mTOR, pathways. AKT/mTOR was involved in a number of important cellular processes including cellular survival and tumor metastasis pathways [51], which is frequently activated in RCC patients [6]. Consistently, we found that the AKT/mTOR pathway is activated in both renal cancer cell lines, suggesting that the activation of the AKT/mTOR pathway is a common event in renal cancer. In our study, we found simvastatin could significantly suppress the phosphorylation/activation of AKT, as well as inhibited its downstream effectors, mTOR. To further investigate whether the pro-apoptotic and anti-metastatic effects are mediated by targeting AKT/mTOR pathway, we applied siRNA to knockdown the expression of AKT. We found that inhibition of AKT activation by knockdown of AKT expression sensitized renal cancer cells to simvastatin-induced anti-cancer effect. Furthermore, it has been reported that AKT exerts its biological effect by phosphorylating its downstream substrates including mTOR. Phosphorylated mTOR becomes activated, leading to increased proliferation, motility and survival of cancer cells [16]. In addition to inhibition of AKT activity, we found that simvastatin treatment can inhibit mTOR phosphorylation. Thus, these data suggest that simvastatin may inhibit the AKT/mTOR axis to induce anti-cancer effect. It is noteworthy that the dose at which statins enhance AKT activation and survival in endothelial cells is the same dose that inhibits AKT activity in cancer cells [52]. This character of statins will be extremely crucial in avoiding side effects when stains are used as cancer therapeutics.

In the last decade, tumor-associated inflammation has become the focus of attention with good reason [53]. Inflammation can contribute to multiple hallmark capabilities of cancer by supplying bioactive molecules to the tumor microenvironment, including inflammatory cytokines, and inductive signals that lead to cell proliferation and metastasis [54]. Signal transducer and activator of transcription (STAT) proteins play central role in regulating cytokine-dependent inflammation and immunity. Amongst all the STATs signals, persistently activated STAT3 via IL-6 induced JAK2 activation promotes cancer cell proliferation, survival and invasion by promoting pro-oncogenic inflammatory pathways [55].

IL-6 is one of the most common cytokines in cancer, and it is overexpressed in patients with metastatic renal carcinoma [56]. IL-6 has been found to play important role in cell migration, invasion, proliferation, apoptosis, angiogenesis and differentiation in cancer cells [57]. STAT3, a new oncogenic target in the setting of renal cell carcinoma, plays a critical role in promoting cell proliferation, metastasis and angiogenesis. Accumulating clinical data suggest that human RCC cell lines are sensitive to JAK2/STAT3 pathway inhibitors. Moreover, the first-line targeted drug sunitinib has recently been shown to elicit its anti-tumor activity by STAT3 inhibition.

To date, there is no data on the effects of simvastatin on regulating IL-6-induced JAK2/STAT3 signaling in renal cancer cells. For the first time, we found that simvastatin could significantly inhibit IL-6 induced proliferation and metastasis of RCC cells, attenuate IL-6-induced JAK2 activation, and subsequently decrease the phosphorylation of STAT3 both *in vitro* and *in vivo*. Further knockdown of STAT3 with siRNA showed significantly decreased cell proliferation and mobility. All these findings suggest JAK2/STAT3 pathway may play a crucial role in simvastatin-induced anti-tumor effects in RCC cells.

Taken altogether, our study demonstrates that simvastatin can markedly exhibit pro-apoptotic and metastasis inhibitory effect on RCC cells. Mechanically, we found AKT/mTOR, ERK, as well as JAK2/STAT3 pathways account for the anti-tumor effects of simvastatin. All these results imply that simvastatin may be a promising therapeutic drug for renal cancer patients.
